# Methylation-Mediated Molecular Dysregulation in Clinical Oral Malignancy

**DOI:** 10.1155/2012/170172

**Published:** 2012-05-07

**Authors:** Rebecca Towle, Cathie Garnis

**Affiliations:** ^1^Department of Integrative Oncology, British Columbia Cancer Research Centre, 675 West 10th Avenue, Vancouver, BC, Canada V5Z 1L3; ^2^Division of Otolaryngology, Department of Surgery, Faculty of Medicine, University of British Columbia, 910 West 10th Avenue, Vancouver, BC, Canada V5Z 4E3

## Abstract

Herein we provide a concise review of the state of methylation research as it pertains to clinical oral cancerous and precancerous tissues. We provide context for ongoing research efforts in this field and describe technologies that are presently being applied to analyze clinical specimens. We also discuss the various recurrent methylation changes that have been reported for oral malignancy (including those genes frequently silenced by promoter methylation and the small RNAs with activity modulated by methylation changes) and describe surrogate disease markers identified via epigenetic analysis of saliva and blood specimens from patients with oral cancer.

## 1. Background

Oral cancer remains a major global killer [1]. Its entrenched poor survival rates make essential the ongoing molecular evaluation of oral cancers and precancers for the purpose of uncovering new tools for detecting and treating this disease. As with most solid epithelial tumors, oral cancers develop through a series of histopathological stages; hyperplasia leads to various degrees of dysplasia, which are followed by carcinoma in situ and finally invasive disease stages. The accumulation of various genetic and epigenetic alterations is understood to drive this progression paradigm. Herein we will discuss the role of DNA methylation in clinical oral tumorigenesis, focusing specifically on oral squamous cell carcinomas (OSCCs).

DNA methylation occurs most frequently at cytosine residues of CpG dinucleotides in gene promoter regions, and much less frequently, within a gene [2]. CpG islands (CpG-rich regions spanning >500 bp with >55% GC content) exist in approximately 60–70% of promoters in the human genome [2, 3]. Methylation in the promoter region of a given gene can serve to decrease expression of that gene. This is thought to occur by either physically inhibiting the binding of proteins essential for transcription, or by recruiting proteins that have transcription repressive properties [4]. This reversible process helps govern gene expression activity in individual cells and is commonly disrupted in cancer, where gene silencing via methylation in particular can contribute to Knudsonian two-hit disruption of tumor suppressor genes [5].

Further, global hypomethylation of genes is understood to serve as a mechanism of oncogene activation, providing another avenue for methylation changes to contribute to tumorigenesis. In addition to being identified as an early event in tumorigenesis for many epithelial cancers, aberrant methylation has also been identified in dysplasia—and tumor—adjacent “normal” tissues, indicating additional complexity at this level of epigenetic dysregulation.

## 2. Techniques and Methodologies for Evaluating Methylation Changes

Myriad technologies exist for detecting DNA methylation alterations and most of these have already been applied in an oral cancer context. The most common technique is methylation-specific PCR (MSP). This approach involves bisulfate conversion of unmethylated cytosines within CpG islands of the genome, with methylated cytosines unchanged by this conversion. In MSP, following design of primers for both methylated and unmethylated sequences of a specific locus, converted DNA is amplified and then separated by gel electrophoresis. Differential analysis of resultant MSP products—say for tumor versus patient-matched normal tissues—reveals changes in methylation status. MSP is particularly useful because it has sufficient sensitivity to detect one methylated cytosine in 1000 and the primers used are also highly specific (i.e., have a low false positive rate). MSP is also a relatively quick and affordable technique, though its limitations include the reality that it is more qualitative than quantitative and the fact that it is generally unhelpful for the assessment of genome-wide methylation changes.

Other techniques for evaluating methylation changes are built on the principles underpinning MSP. MethyLight—which integrates sodium bisulfite conversion and quantitative fluorescence PCR—offers a sensitive, highly specific, and rapid means for assessing methylation status for a particular locus [6]. This technique involves use of primers with a fluorescent 5′ reporter dye (typically FAM) and a 3′ quencher dye. During amplification, Taq DNA polymerase cleaves the probe and releases the reporter dye. The resultant fluorescence is quantified by laser in associated equipment and will be proportional to the number of methylated cytosines at a given locus. This approach allows a more precise quantitation of methylation status compared to MSP, which can only be measured qualitatively. Additionally, MethyLight has been shown to be more sensitive than MSP by a factor of ten (detecting even one methylated cytosine in 10,000) [6].

Sequence alterations resulting from bisulfite conversion can remove cut sites recognized by specific restriction enzymes. For example, if methylation has been lost, the cut site for *Bst*UI may be lost via conversion of a CGCG sequence to TGTG. COBRA (combined bisulfite restriction analysis) leverages this fact to allow quantification of methylation in PCR products generated following bisulfite conversion [7]. Ultimately, the amount of total methylation per locus corresponds to the degree to which the strands are cut by the restriction enzyme. The strength of this technique lies in its quantitative results, ease of use, and compatibility with paraffin embedded samples. However, COBRA does have its limitations, as it is a locus specific technique and thus unhelpful for a genome wide analysis.

Pyrosequencing is a technique that is quantitative, easy to use, and accurate [8]. It utilizes a sequence-by-synthesis method that monitors the bioluminometric signal that results when a pyrophosphate group is released during DNA synthesis. This technique can assess the methylation status of genes by bisulphate conversion, PCR, and subsequent comparison of the ratio of T and Cs in the samples.

A recurring disadvantage to the above techniques is the fact that they do not easily facilitate global analyses of methylation changes; they are all locus-specific. Several techniques for global evaluation of changes in the methylome have been developed to address this issue. Currently, the most common approach for genome-wide analysis involves methylation-specific microarrays. A range of arrays are available, but most are based on one of three general principles to sample processing: (MSRE) methylation-sensitive restriction enzyme digests, methylation-specific immunoprecipitation, or sodium bisulfite conversion [9].

Regarding enzyme digestion approaches, sample processing with endonucleases specific to methylation sites (e.g., *HpA*1I) was one of the first methods of array-based methylation profiling [10]. There are several different types of arrays that use this approach. For example, in differential methylation hybridization arrays, DNA is digested by MSREs. This pool of DNA fragments is labeled with one fluorescent dye, and a pool of the same sample with no digestion is labeled with second dye. They are concurrently hybridized on an array and relative signal intensities are measured to determine methylation status at a given locus [10]. This method of methylation profiling can interrogate everything from hundreds of different CpG islands to the entire genome. These approaches all have relatively high sensitivity; however, their utility for analyzing disease tissues can be variable as DNA sample input requirements can be quite high, requiring approximately 2 *μ*g of tissue, and clinical lesions—particularly premalignant ones—can be quite small (thus limiting the amount of specimen available for analysis). Additionally, these methylation arrays can be prone to false positives, have a low throughput as only several samples can be processed in parallel, and analysis is limited to those sequences that possess recognition sites for the restriction enzyme associated with a given platform [9].

Immunoprecipitation of either methylated DNA or chromatin, known as MeDIP and ChIP, respectively, has also been used to facilitate global analyses of methylation alterations [11, 12]. These methods use antibodies to pull down material of interest and subsequently subject it to microarray analysis. This method has the ability to detect methylation status at a genome-wide level, does not require specific primers, and avoids the sequence bias introduced by restriction enzyme methods [9]. However, this technique can also have lower throughput than other approaches and has low sensitivity to methylated areas that occur in CpG poor regions. Additionally, instead of microarray analysis of pulled down material, ChIP-Seq combines immunoprecipitation with sequencing technology to elucidate protein-DNA interactions on a genome-wide scale [13].

A third common approach to methylation-specific microarrays involves use of the sodium bisulfite conversion methods described above. Illumina arrays offer one platform based on this approach [14]. These arrays are automated in use and allow analysis of hundreds of samples simultaneously. They are also capable of delineating methylation status for thousands of CpG sites (if not the entire genome), allow determination of the methylation status of specific CpG sites, and have relatively low sample input requirements (thus more readily facilitating analysis of low yield clinical tissues). That said, this technique can introduce bias via incomplete bisulfite conversion reactions and PCR.

Lastly, a technique that is rapidly becoming increasingly common is whole genome bisulfite sequencing. Due to improvements in protocols, increase of throughput, and the rapid decline of sequencing cost, this method is robust and highly sensitive. In brief, this protocol involves bisulfite conversion, PCR amplification, and sequencing of products to determine methylation status to a single base pair resolution [15]. Historically, this method was problematic due to processing errors of bisulfite conversion, degradation of DNA due to long processing times, and the need for a relatively large amount of sample [16]. However, improvements on protocols have greatly increased efficiency of this technique, which has great promise for the profiling of methylation status.

## 3. Genes Evincing Methylation Changes in Oral Squamous Cell Carcinomas

As specific genes are implicated as critical drivers of malignant phenotypes, they become attractive candidates for evaluation as targets of novel therapeutics or as biomarkers for guiding patient management decisions. Recurrent methylation-mediated alterations of several genes have been reported for all invasive epithelial cancer types, including oral tumors. It is understood that in general cancer cells exhibit increased global hypomethylation with specific regions of hypermethylation. However, it is currently not known how specific genes are targeted. The major etiological factors for oral squamous cell carcinoma (OSCC) are smoking, alcohol use, and (to a lesser extent) Human Papillomavirus (HPV) infection [17]. How these factors influence DNA methylation in oral tumorigenesis has not been evaluated. However, smoking has been shown in other cancer types to activate DNA methyltransferase 1 (DNMT1) which catalyzes DNA methylation [18, 19]. A summary of specific genes implicated in oral cancers and understood to be governed by methylation changes follows below.

*CDKN2A*, mapping to chromosome 9p21.3, produces two major proteins: p16(INK4), which is a cyclin-dependent kinase inhibitor, and p14(ARF), which binds the p53-stabilizing protein MDM2 and is involved in cell cycle control. Deletion at this locus is regularly reported to be one of the earliest events in oral cancer initiation and progression [20]. Hypermethylation of the *CDKN2A* promoter region has been extensively evaluated in oral cancers with the frequency of hypermethylation being reported as anywhere from 28% to 86% [21, 22]. Aberrant methylation of this locus in noncancer controls has not been detected [23–25]. A panel of cell lines was investigated for homozygous deletion, hypermethylation, and point mutations at *p16*, with results indicating that the first two alteration types were the more common modes of *p16* disruption in OSCC [26].

In a specific cohort of betel chewing individuals with oral cancer, methylation of *p16* was detected in 63% of OSCCs and 67% of verrucous carcinomas [27]. In a panel of individuals of Indian descent, methylation of *p16* was detected in 23% of OSCC cases [24]. In general, data do suggest that differences in patient ethnicity, etiological factors, and tissue type (since the OSCC category actually spans different tissues) can influence the molecular alterations detected for disease.

Several groups have correlated methylation of *p16INK4A*/*p14ARF* with various clinical features for oral cancers, though results have varied. In one study, it was observed that people with *p16* promoter methylation had a lower mean age, a higher risk of lymph node invasion in young patients, a higher risk of distant metastasis in older patients, and shortened disease free survival in older patients [28]. Other work showed that *p16INK4A* methylation was associated with increased likelihood of disease recurrence, whereas *p14ARF* is associated with lower recurrence rates [29]. Concurrent promoter hypermethylation of *p16* and *p14* correlated significantly with tumor size and lymph node metastasis and with later stage of OSCC in one study [30], while a separate study found methylation of *p14ARF* alone correlated with a good prognosis for patients [31]. Larger scale trials are needed to fine tune how methylation status of *p16* and *p14* promoters may best be applied to manage clinical decisions.

*P16* promoter methylation has been assessed for squamous cell carcinomas of the tongue as well as in margin tissues that remain in patients following surgical resections. As expected, tumors showed a high frequency of *p16* promoter hypermethylation (86.8%) [32]. Regarding tissues at surgical margins—which were all histologically-characterized as disease-free—43.3% exhibited *p16* promoter hypermethylation [32]. Significantly, those cases with margin tissues harboring *p16* hypermethylation had a 6.3-fold increased risk for local recurrence.

Similarly, a separate group assessed *p16* promoter hypermethylation status in OSCC tumors, associated normal tissues, and a panel of healthy controls [33]. They found no methylation of *p16* in the healthy control group whereas *p16* methylation in OSCC-associated normal tissues was detected for 27.3% of cases (and in all of those cases, concurrent *p16* hypermethylation was also detected for matched tumors). In this study, clinical features and habitual factors did not correlate with methylation status. Another study did not report prognostic significance for *p16* methylation, again showing that there is lingering ambiguity regarding whether *p16* hypermethylation will have utility as a clinical biomarker [34].

*E-cadherin* (*CDH1*) plays a critical role in cell adhesion processes and is known to significantly influence epithelial tissue architecture [35]. With respect to malignancy, it is known to function as a suppressor of invasion and metastasis formation and has previously been reported as undergoing hypermethylation-mediated silencing in several cancer types [35]. Previous studies of tongue squamous cell carcinoma revealed that downregulation of *CDH1* expression via promoter hypermethylation was significantly associated with poorer rates of disease-free survival [36]. Independent reports have confirmed association of epigenetically-silenced *CDH1* expression and poorer overall survival for oral cancer (also demonstrating that *CDH1* promoter hypermethylation is associated specifically with poorer survival for node-positive cases and individuals with stage III disease) [34]. Other groups, on the other hand, while reporting associations between *CDH1* promoter hypermethylation and oral cancers (when compared to normal oral mucosa), have failed to detect significant associations between these same clinical parameters and this epigenetic event [28]. Independent evaluation based on a large OSCC patient cohort is needed to more accurately determine the significance of the methylation status of *CDH1* vis-à-vis clinical outcomes (something that can also be said for other genes reported as epigenetically dysregulated in oral tumors).

*O* ^6^ *-methylguanine-DNA methyltransferase* (*MGMT*) is a DNA repair gene that protects from toxicity and mutations that occur by alkylating agents through the removal of O^6^-guanine DNA adducts. CpG island hypermethylation of the *MGMT* promoter region results in gene silencing, with loss of MGMT repair capacity thought to drive cancer progression via the emergence of genomic instability. Decreased expression of *MGMT* via epigenetic silencing has been reported for many cancer types and loss of its expression can be tied to greater sensitivity to alkylating chemotherapeutic agents [37]. Epigenetic silencing of MGMT has been associated with OSCCs where tobacco exposure and betel quid chewing are suspected etiological factors [38–40]. MGMT promoter hypermethylation has also been associated with poorer outcomes for oral cancer, including a greater likelihood of nodal metastases, tumor recurrence, and decreased survival [41, 42]. MGMT promoter hypermethylation has also been associated with poorer outcomes for oral cancer, including a greater likelihood of nodal metastases, tumor recurrence, and decreased survival [32, 33]. Reduced MGMT expression has also been associated with these parameters in head and neck squamous cell carcinomas generally, and OSCCs specifically [41, 43]. Ongoing or elevated MGMT expression has been associated with resistance to alkylating agents in multiple cancer types including gliomas, astrocytomas, and melanomas [44, 45]. While alkylating agents such as ifosfamide and cyclophosphamide have been applied to manage various stages of oral and other head and neck malignancy, we have not found any reports to date regarding the role of MGMT silencing in modulating response to these compounds in these cancer types [46, 47]. Existing data from other cancer types provide a strong rationale for pursuing such studies.

Death-associated protein kinase (DAPK) encodes a serine/threonine kinase that is required for apoptosis induced by IFN-*γ* [48]. Loss of its expression via promoter hypermethylation has been associated with formation of metastases and advanced disease stages in multiple cancer types, including head and neck cancers [38, 48, 49]. Regarding OSCCs, *DAPK* hypermethylation has been reported as associated with increased likelihood of lymph node involvement, though these results have not always attained statistical significance [34, 50]. Interestingly, other groups have not reported associations between these clinical features and hypermethylation of *DAPK* (or other genes discussed here) [42, 51]. Again, this may be a function of the tissue heterogeneity that exists within the oral squamous cell carcinoma category. Detection of *DAPK* promoter hypermethylation at resection margins of oral tumors has been significantly associated with decreased overall survival, suggesting that it may have utility as a biomarker for guiding patient follow-up strategies [52]. As with *MGMT*, *DAPK* hypermethylation has also been associated with oral tumors where tobacco consumption is a suspected etiological factor [39].

The *TGF*β** superfamily transcription factor, *runt-related transcription factor 3* (*RUNX3*), functions as a tumor suppressor gene and is involved in mediating apoptotic processes [53]. Promoter hypermethylation-mediated silencing of *RUNX3* has been reported for many cancer types [53]. Recently, hypermethylation of the *RUNX3* promoter region was found to be significantly associated with the presence of lymph node metastases and tumor stage in tongue carcinomas [52]. Other groups have reported that reduced *RUNX3* expression or promoter hypermethylation is associated not only with progression in oral cancers, but also with disease recurrence and poorer prognoses [52]. The fact that other groups have not found significant associations between *RUNX3* promoter hypermethylation and patient outcomes suggests that the role of epigenetic silencing of this gene in oral cancers bears further scrutiny [54]. The emerging role of *RUNX3*-mediated perturbation of the canonical Wnt signaling pathway in oral cancers also needs to be further evaluated.

### 3.1. Wnt Pathway Genes

 Dysregulation of the canonical Wnt signaling pathway—via disrupted function of genes such as *adenomatous polyposis coli *(*APC*)*, AXIN1, *β*-catenin *(*CTNNB1*), and* secreted frizzled-related proteins *(*SFRPs*)—has been noted for a variety of cancer types, including oral malignancies [55–58]. Briefly, canonical Wnt signaling involves stabilizing CTNNB1. When the canonical Wnt pathway is inactive, CTNNB1 exists in a phosphorylated form and is marked for degradation, with this phosphorylation mediated by a protein complex that incorporates Glycogen Synthase Kinase 3*β* (GSK3*β*), APC, and AXIN proteins. When Wnt binds to receptors encoded by *Frizzled* (*Fz*) genes, Dishevelled (Dsh) is activated and, consequently, GSK3*β* is inhibited. This in turn causes CTNNB1 dephosphorylation, which stabilizes the molecule and allows it to accumulate in the cell nucleus, where it can induce TCF/LEF-mediated transcription of several target genes. Regarding oncogenic processes, the downstream effects of CTNNB1 activation via Wnt signaling include enhanced cell proliferation and antiapoptotic activity.

Given their role as Wnt antagonists, *SFRPs* function as tumor suppressors. Marsit et al., reporting on head and neck cancers in general, described methylation differences for *SFRP* genes based on alcohol consumption, smoking behaviors, and HPV16 status [59]. More specifically, multiple reports involving analysis of both oral cancer cell lines and clinical OSCC cases have reported that promoter hypermethylation for SFRPs is associated with disease [60, 61]. Interestingly, the findings in these studies for *SFRP1* have been conflicting, with both hypermethylation and demethylation of this gene reported [60–62].

*APC* also functions as a tumor suppressor gene and has also been reported as downregulated in oral tumors. Disruption of *APC* function in OSCC has been attributed to loss of heterozygosity (LOH alterations), mutations, and epigenetic alterations [63]. Regarding the latter, *in vitro* studies demonstrated that treatment with demethylating agent 5-aza-2′-deoxycytidine resulted in restoration of *APC* expression in oral cancer cells, supporting the functional importance of epigenetic silencing for this gene [64]. The drug received FDA approval for the treatment of myelodysplastic syndrome and is currently in clinical trials testing its utility in the treatment of several solid tumors. Increased promoter methylation for *APC* has been reported in clinical oral cancer tissues and some data do suggest a relationship between *APC *methylation status and development of lymph node metastases when it is analyzed in concert with the promoter methylation status of *CDH1* [34].

As described above, activation of *CTNNB1* is a critical consequence of canonical Wnt pathway signaling—and a variety of oncogenic processes can be turned on by this activity [56]. Elevated expression and greater nuclear localization of CTNNB1 have been reported for oral cancer and a multitude of other cancer types [56, 65]. While recent work has shown CTNNB1 immunostaining levels to be significantly associated with lymph node status, survival outcomes, and different invasive stages for oral cancers, data regarding the association of *CTNNB1* methylation status for this disease have not [66]. Data from other cancer types suggest that activating mutations of *CTNNB1* that prevent its downregulation may be a more common event than methylation [67].

*WIF1* functions as a tumor suppressor that inhibits Wnt signaling through direct interaction with Wnt proteins, its activation leading to cell cycle arrest [56]. It has been described as downregulated in several cancer types [56]. Promoter hypermethylation-mediated downregulation of *WIF1* in oral cancers has been reported by multiple groups, so there is evidence to suggest that epigenetic alteration of this gene can contribute to invasive disease phenotypes [52, 61]. More recently, a group studying tongue cancers found no significant associations between *WIF1* promoter hypermethylation and lymph node metastasis formation, tumor stage, or overall survival [52]. In a separate study, this same group found no significant association between WIF1 promoter hypermethylation status in tissues from histologically negative resection margins and oral cancer recurrence [52]. Results so far suggest that WIF1 alterations may only have clinical utility as oral cancer biomarkers where used for diagnosis of invasive disease.

**Figure 1:**
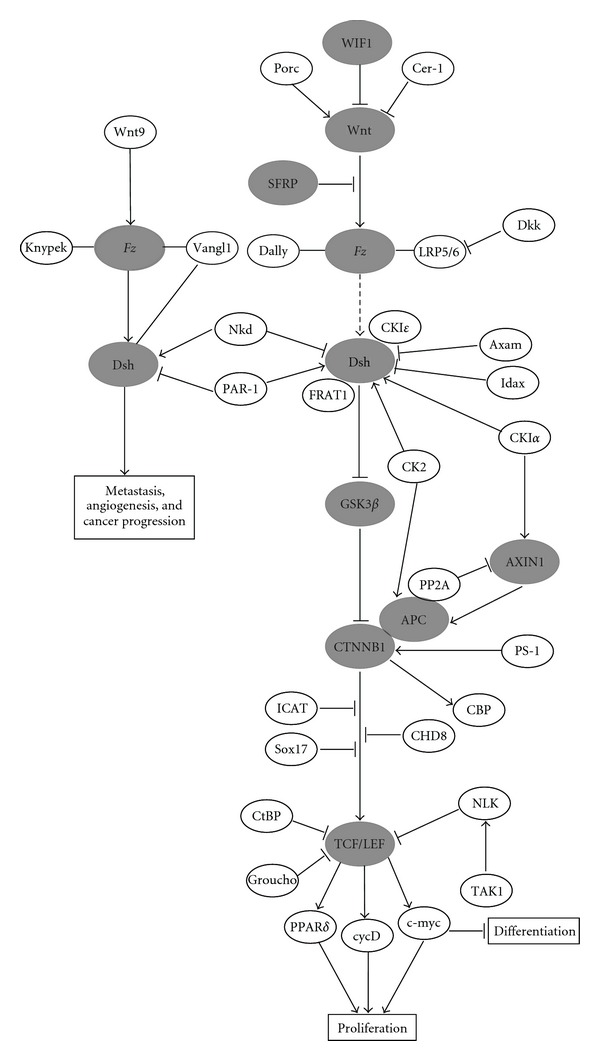
An overview of the canonical WNT signaling pathway. Genes highlighted in this paper as implicated in OSCC are shaded grey. Gene interactions are shown as activating (arrows) or inhibiting (blocked arrows). Boxes indicate processes that will ultimately be influenced by WNT signaling. Solid lines represent direct interactions and dashed lines indicate indirect effects. Lines connecting genes represent binding or association interactions. Complexes are represented by ovals that overlap.

Figure 1 places the genes discussed in this section in their proper context in the canonical Wnt signaling cascade. 

## 4. Methylation Changes Governing Behavior of Noncoding RNAs in Oral Cancers

Noncoding RNAs, particularly microRNAs (miRNAs), have been shown to play a role in many biological processes and cellular pathways—and their behavior has been shown to be modulated by methylation changes [68, 69]. MiRNAs are 19–24 nucleotide transcripts that regulate mRNA expression by binding to and subsequently silencing the mRNA target [70–72]. MiRNAs can act as oncogenes or tumor suppressors and have been identified as dysregulated in several cancer types, including oral cancer [73–77]. As is the case with protein coding genes, there are several mechanisms that can lead to abnormal miRNA expression, including hypermethylation of CpG island promoters [78–82]. Using a functional-based screen in two OSCC cell lines, Uesugi et al. profiled tumor suppressive miRNAs that are silenced by hypermethylation [76]. They identified 110 miRNAs that exhibited inhibitory properties and, when compared to additional cell lines and tumor tissue, they found that miR-218 and miR-585 were frequently silenced by DNA hypermethylation [76].

Separate work has also shown that silencing of four tumor suppressive miRNAs (miR-34b, miR-137, miR-193a, and miR-203) can be mediated by aberrant methylation in OSCC cells [68]. This same work reported downregulation of miRNA expression through tumor-specific hypermethylation as being more frequent for miR-137 and miR-193a than for miR-34b and miR-203 when analyzed in primary tumors and paired normal oral mucosa. A separate group investigated the association between promoter methylation of miR-137 and both overall survival and disease-free survival, as well as with various prognostic factors [83]. This study found an association between methylation at the miR-137 promoter and poorer overall survival, though no associations were observed with disease-free survival or any of the other evaluated prognostic factors. MiR-137 has been reported as a negative regulator of CDK6/CCND1-mediated cell cycle progression [84], hence promoter methylation might serve as a means of inactivating tumor suppressive miR-137 function. Langevin et al. suggest that their inability to detect associations between miR-137 and other prognostic features in their study may have been a product of factors such as limited sample sizes and insufficiently long followup, proposing that stronger associations between miR-137 and oral cancer outcomes may exist [83]. Very recent work has suggested that epigenetically-mediated miR-137 expression may differ in oral cancer cells depending on whether they are part of a stem-like subpopulation [85]. The argument for further evaluation of miR-137 in the context of oral cancer outcomes is bolstered by evidence from other cancer types that also suggest a tumor suppressive biological role for this miRNA and an association with poorer disease outcomes [86–88]. Though only a few reports have evaluated the impact of methylation changes on small RNA behaviors in oral cancers and precancers, these early data do suggest a role for these molecules in oral malignant processes and provide a strong rationale for further studies in this area.

## 5. Altered Methylation States in Oral Premalignant Lesions and Cancer Progression

Premalignant lesions in the oral cavity are readily detectable owing to the accessibility of the organ site. That said, one factor that contributes to the poor prognosis of OSCC is the current inability to determine which premalignant lesions will ultimately progress to invasive disease; the clinical standard of care is “watchful waiting” since histopathological review is presently incapable of delineating progression risk and intervention with all early lesions would lead to overtreatment that is costly both in terms of dollars and patient quality of life. Since earlier stage lesions are smaller in size and more readily treatable, biomarkers delineating the progression likelihood for a given dysplasia case represent a key means for improving patient outcomes.

Several studies have indicated that deletion and silencing of loci mapping to chromosome arms 9p and 3p are common events in the early development of OSCC [89, 90]. Thus, studies investigating the role methylation changes in oral premalignant lesion (OPL) behavior have generally investigated genes mapping within these regions.

More specifically, several studies have investigated the impact of methylation changes *p14* and *p16*—both of which, as described above, are encoded by *CDKN2A* and map to chromosome 9p21.3—in OPLs. A much higher degree of *p16* promoter hypermethylation has been reported relative to *p14* promoter hypermethylation in patients with histologically confirmed severe dysplasias (57.5% and 3.8% resp.) [91]. Another group reported that *p16* hypermethylation in OPLs was associated with a greater risk for progression to invasive disease; patients with confirmed OPLs (as determined by WHO criteria and independent review by two pathologists) that harbored hypermethylated *p16* promoters were approximately two and a half times more likely to develop OSCC than patients with OPLs that did not exhibit *p16* promoter hypermethylation [92]. Interestingly, another study reported higher rates of promoter methylation for neighboring *p15* relative to *p16* across multiple stages of histopathologically confirmed oral dysplasia (50% versus 18%) [93]. This same study also reported a much higher frequency of *p14* promoter methylation; though the fact that analyzed tissues came from a population of betel quid chewers—where the suspected disease etiology was therefore different than other populations (where tobacco is the predominant etiological factor)—may have impacted these results.

Another study assessed whether hypermethylated genes previously implicated in oral dysplasias could be used as markers for progression likelihood [94]. In this instance, longitudinal follow-up data made this evaluation possible since progression status for each patient with clinically confirmed disease was known. Interestingly, the results of this work suggested a much lower incidence of promoter methylation at previously identified candidate genes than in the studies discussed above. The authors in this instance posited that use of pyrosequencing instead of MSP may explain the discrepancy, the former technique being less prone to false positives. This study did conclude that *p16* could be a predictor of progression, though its specificity came in at a fairly low value (57%).

Though wealth of clinical data associated with canonical genomic alterations at chromosome 9p and 3p makes these regions attractive for locus-specific analysis of methylation alterations, other loci are also worthy of further scrutiny for the utility in predicting disease progression. *14-3-3-*σ** has also found to be recurrently methylated in histologically confirmed oral dysplasias and has been associated with coincident methylation at *p16*, making it an attractive candidate for further evaluation in an oral cancer progression context [95]. Another group observed that *RECK*, a gene that functions to inhibit angiogenesis, invasion, and metastasis, has a potential to be a biomarker as it is recurrently found to be hypermethylated in the normal mucosa adjacent to the tumor [96]. Ultimately, whole methylome analyses of a large panel of well-annotated OPLs with extensive followup are needed to uncover disease-relevant biomarkers that will impact disease management and oral cancer survival rates.

## 6. Global Methylome Changes and the Role of Hypomethylation in Oral Malignancy

In addition to investigating the methylation status of specific genes, some studies have begun analyzing the oral cancer methylome as a whole. Early evidence suggests that the distribution of methylation at various gene promoters across a series of lesions of progressing grade from a single individual can follow a specific pattern. Further, the CpG island methylation phenotype (CIMP) has been observed in several cancer types and is based on the observation that those tumors exhibiting aberrant methylation of one gene are more likely to have other sites of aberrant methylation [97, 98]. To determine whether the CIMP phenomenon was detectable in OSCC, the methylation status of ten genes was evaluated in a large panel of oral tumors [99]. This analysis revealed a cluster of tumors with a greater degree of promoter methylation than would be predicted by chance alone. These cases were identified as “CIMP +ve” and results suggest (1) that these tumors exhibited a less aggressive tumor biology than other oral cancers and (2) that these cases were characterized by a greater host inflammatory response (a finding which the authors note is consistent with findings in other cancer types). Another group has reported observing CIMP in head and neck cancers, though it is clear additional studies are needed to elucidate the mechanisms driving this phenotype [100].

While the majority of literature has focused on promoter hypermethylation silencing of tumor suppressor genes as a critical mechanism driving oral tumorigenesis, DNA hypomethylation is also understood to contribute to development of many epithelial cancers by facilitating activation of candidate oncogenes [101]. More specifically, it has been proposed that DNA hypomethylation contributes to tumorigenesis by two potential mechanisms. The first represents another global level of methylation dysregulation, where highly methylated repetitive elements (such as long interspersed nuclear element-1 LINE-1 and *Alu *sequences) are demethylated, resulting in increased chromosomal instability that leads to irregular mitoses that, in turn, drive the emergence of further mutations that can activate oncogenes [97, 102, 103]. The second is more specific, where inadvertent demethylation in a local context activates oncogenes that are normally methylation-silenced in the human genome [97, 102, 103].

With respect to oral cancer specifically, little work has been done in clinical tissues. Analysis of *in vitro* OSCC models of interleukin-mediated chronic inflammation have indicated that chronic inflammation can drive both global hypomethylation of LINE-1 sequences as well as specific CpG methylation changes [104]. More precisely, this model showed that IL-6 specifically was inducing these changes, with methylation alterations also found to be associated with downstream changes in gene expression. The suggestion that inflammatory responses can play a critical role in mediating cancer-causing methylation changes is of particular interest given that the oral cavity, which experiences regular exposure to carcinogens, is frequently under inflammatory stress. Another group investigated the role of hypomethylation in a murine model of oral cancer which used *DNMT1* hypomorphic alleles to reduce genomic methylation as lingual and esophageal carcinogenesis was induced by 4-nitroquinoline 1-oxide [105]. This group reported that reduction of DNA methylation levels led to the suppression of tumor formation in certain cell types.

A separate group detected global LINE-1 hypomethylation in oral rinses obtained from OSCC patients (relative to healthy controls) [106]. This is touched on further in the following section. The relative scarcity of studies addressing either global trends in the oral cancer methylome or the role of specific instances of hypomethylation in oral tumorigenesis points to a need to develop these research streams more fully.

## 7. Methylation Changes as Surrogate Markers for Oral Malignancy

### 7.1. Saliva

The use of saliva for the diagnosis or establishing prognosis of OSCC patients is a promising screen, as it is both noninvasive and inexpensive. The potential of this tool has been studied by utilizing a genome-wide methylation array to profile the methylation status of 13 OSCC patients before and after surgery, as well as ten normal samples [107]. This work identified 34 genes of interest, including *p16*, and proposed panels ranging from 4–7 genes that are likely to have the optimal sensitivity and specificity for clinical applications (the range of specificity being 62–77% and sensitivity being 83–100%). This group also used saliva samples taken from the same patients at both the pre- and postoperative stages, eliminating issues arising from using saliva samples from unmatched normal subjects as a control.

Another group analyzed the methylation status of 11 genes by MSP for a panel of primary tumors and saliva samples from 90 patients with HNSCC [108]. They found several genes that were frequently methylated in the paired saliva and tissue samples including previously-implicated candidates such as *p16*, *MGMT*, *DAPK*, and *RASSF1*. Additionally, they were able to accurately detect malignant cells in saliva several months before tumor recurrence was otherwise detected in patients, indicating that saliva-based assays may be a very useful tool for effective followup of oral cancer patients.

As referenced above, a separate study of oral rinses identified LINE-1 hypomethylation as being more prevalent in rinses obtained from OSCC patients when compared to healthy controls [106]. While the authors did not establish any statistically significant associations between their rinse results and disease stage, histological grade, lesions site, or carcinogen exposures (e.g., tobacco smoke), the utility of this noninvasive approach for differential diagnosis of oral malignancy does suggest it is worth evaluating in independent patient cohorts.

To assess the potential of saliva as a screen for premalignant lesions, another group collected saliva samples from patients that had leukoplakia and were at risk for the development of OSCC [109]. They assessed the methylation status of *p16*, *p14,* and *MGMT* from these specimens using MSP and observed a relatively high frequency of hypermethylation of *MGMT* and *p16* relative to levels typically found in OSCC patients. However, followup had not been done on the patients, so whether or not the hypermethylation of *p16* and *MGMT* was unique to patients at risk for progressing into OSCC is not known.

### 7.2. Serum

In addition to saliva rinses, methylation changes detected in DNA isolated from serum from peripheral blood also hold potential as a diagnostic or prognostic tools for detection and management of disease. This approach has previously been used in a variety of other cancers such as lung, liver, and colorectal [110–112]. As with studies involving saliva, the methylation status of *p16* has been the predominant focus of this work in oral cancer.

One group found that parallel evaluation of promoter methylation status of multiple genes (including *p16*) improved the sensitivity and specificity of disease discrimination [113]. Another group similarly analyzed the promoter methylation status of multiple genes in serum samples obtained from OSCC cases and found that in a majority of cases, at least one of the four assayed genes was detectable as hypermethylated [49]. It has been reported that when a gene is found to be hypermethylated within a tumor, DNA from a matched peripheral blood sample will harbor similar methylation changes at a frequency ranging from 31 and 54.5% [49, 114, 115].

Evaluation of methylation alterations in samples obtained by minimally invasive procedures such as collection of saliva or blood remains a very attractive avenue for the discovery of biomarkers for management of clinical oral malignancy. Expanded collection efforts and evaluation of these biological materials should be a component of all molecular investigations of oral cancers and precancers.

## 8. Conclusion

Interrogation of methylation changes in clinical oral cancers and precancers has revealed multiple recurrent alterations at genes and chromosomal loci that are associated with oncogenic processes in this and other cancer types. Some analyses have indicated strong associations between these changes and specific patient outcomes, including the likelihood of progressing from premalignant to invasive disease and posttreatment recurrence. Evaluation of blood and saliva samples, that are easily accessible, has also generated data that methylation changes detected in these specimens might have utility as surrogate biomarkers for managing disease. Given that oral cancers and precancers represent a significant global health challenge, these more affordably processed specimen types should be of particular research interest going forward if the field is serious about developing tools with utility worldwide. We conclude that changes in DNA methylation from clinical tissues do show early promise as markers to improve management for this cancer type, which has had survival rates that have remained stubbornly low over recent decades. We ultimately feel that the true utility of methylation markers for management of oral malignancy will only be realized when analyses are expanded to much larger patient cohorts. This reasoning holds whether the methylation status is being evaluated for an individual locus or by the most current and robust platform for whole methylome analysis. We also feel that a much stronger research emphasis on developing new methylation markers for managing smaller, more treatable premalignant lesions would address a currently underserved path for improving oral cancer outcomes.
